# Which muscle is the external rotation compensator after superior capsular reconstruction?

**DOI:** 10.1016/j.jseint.2024.09.010

**Published:** 2024-09-27

**Authors:** Nattakorn Mahasupachai, Nobuyuki Yamamoto, Atsushi Arino, Jun Kawakami, Rei Kimura, Toshimi Aizawa

**Affiliations:** a Department of Orthopaedic Surgery, Tohoku University School of Medicine, Sendai, Japan; b Department of Orthopaedics, Faculty of Medicine, Srinakharinwirot University, Nakhon Nayok, Thailand

**Keywords:** Massive irreparable rotator cuff tear, Superior capsular reconstruction, External rotation compensator, Posterior deltoid, Teres minor, Muscle volume study

## Abstract

**Background:**

Superior capsular reconstruction (SCR) is a surgical option for massive irreparable rotator cuff tears, particularly involving the supraspinatus and infraspinatus. In this procedure, the torn infraspinatus is not repaired or reconstructed. However, an improvement in postoperative external rotation (ER) angle and strength is observed. There is a lack of studies explaining how ER is restored after SCR. The aim of this study is to identify the ER compensator by assessing the muscle volume of the posterior deltoid and teres minor.

**Methods:**

Sixty-eight patients with massive rotator cuff tears underwent SCR during 2016-2021. Of these patients, 28 who met the following inclusion criteria were retrospectively reviewed: (1) massive rotator cuff tears, including the supraspinatus and infraspinatus, (2) severe muscle atrophy and fatty change, (3) intact or reparable subscapularis tendon, and (4) Hamada of grade 3 or lower. Posterior deltoid and teres minor volume were measured using open-source medical image processing software preoperatively and 1-year postoperatively. The percentage of the posterior deltoid and teres minor muscle volume change was compared between patients with 1-year postoperative ER manual muscle testing (MMT) of grade 5 and of grade < 5. The relationship between grade of fatty change, percentage of the muscle volume change, and ER angle and strength were evaluated.

**Results:**

There was a significant increase in the 1-year postoperative teres minor volume compared with the preoperative volume (24.6 ± 10.3 cm³ vs. 20.9 ± 8.3 cm³, *P* < .000), while the posterior deltoid volume remained unchanged (178.1 ± 48.3 cm³ vs. 178.8 ± 47 cm³). Patients with ER MMT of grade 5 had a greater teres minor volume change compared to those with an ER MMT grade of less than 5 (22.3% vs. 9.4%), although this difference was not significant (*P* = .074, 95% CI = −1.3 to 27.0). The posterior deltoid volume showed no significant change. The percentage of teres minor volume change had a weak positive correlation with ER strength (r = 0.308, *P* = .055, 95% CI = −0.02 to 1.0). There was a significant negative correlation between ER strength and the severity of both preoperative and postoperative fatty changes in the teres minor (r = −0.258, *P* = .065, 95% CI = −1.0 to −0.042 and r = −0.323, *P* = .028, 95% CI = −1.0 to −0.113, respectively). The pre and postoperative fatty changes in the teres minor were negatively correlated with the percentage of teres minor volume change (r = −0.298, *P* = .062, 95% CI = −1.0 to 0.031 and r = −0.413, *P* = .015, 95% CI = −1.0 to −0.1, respectively).

**Conclusion:**

The teres minor may serve as a potential compensator for ER in patients with massive rotator cuff tears following SCR.

Surgical treatment for irreparable large-to-massive rotator cuff tears is a challenging issue. Treatment options remain controversial, particularly in young patients. Various procedures, such as partial repair, tendon transfer, and reverse total shoulder arthroplasty, aim to alleviate symptoms and restore function.^2^^,^^9^^,^^11^^,^^30^^,^^46^^,^^62^^,^^68^ Recently, superior capsular reconstruction (SCR) was developed as an alternative joint-preserving option for irreparable posterosuperior cuff tears.^44^

A massive rotator cuff tear causes a vertical imbalance in the glenohumeral joint. The humeral head is pulled upward by the deltoid muscle during forward flexion and abduction.^3^ Reconstruction of the superior capsule helps prevent upward migration of the humeral head by restoring superior glenohumeral stability and maintaining the acromiohumeral interval as a spacer.^45^ This novel procedure has yielded excellent clinical outcomes in shoulder function, as reported by various institutions and countries.^44^^,^^56^^,^^57^^,^^60^^,^^63^ Theoretically, SCR does not directly reconstruct or repair the infraspinatus, the most effective external rotator, to its original footprint. Consequently, preoperative external rotation (ER) deficit is often viewed as a significant drawback of SCR compared to tendon transfer.^9^^,^^49^^,^^60^^,^^68^ Contrary to expectations, however, despite the absence of this key external rotator, both ER motion and strength have improved following SCR.^1^^,^^4^^,^^5^^,^^7^^,^^8^^,^^10^^,^^27^^,^^35^^,^^41^, ^42^, ^43^, ^44^^,^^64^^,^^67^ The lack of concrete evidence explains the recovery of ER strength and angle after SCR. One possible reason could be the compensation by the remaining external rotators, such as the teres minor or posterior deltoid.^19^^,^^24^^,^^25^^,^^29^^,^^33^ The hypertrophy of these muscles is reported to be evidence of adaptation and compensation.^28^^,^^29^^,^^38^^,^^50^^,^^61^ This factor might play a significant role in deciding between SCR and tendon transfer.

The aim of this study was to determine whether the teres minor or posterior deltoid is the ER compensator by assessing the muscle volume in the patients who underwent SCR with recovery of ER muscle strength. We hypothesized that, as the compensator, the teres minor or deltoid would increase their muscle volume postoperatively compared to preoperative volume and should have a relationship with ER angle or strength.

## Materials and methods

This study was approved by the Tohoku University Hospital Institutional Review Board (No. 2022-1-549). A retrospective review of the patients’ data between September 2016 and March 2021 was conducted in Tohoku university hospital and affiliated hospitals. Sixty-eight patients who underwent arthroscopic SCR with the following criteria were recruited in this study: (1) patients with posterosuperior large-to-massive rotator cuff tears, including supraspinatus and infraspinatus tendons, (2) severe muscle atrophy (more than medium according to the Warner classification^64^ and fatty change (grade ≥3 according to the Goutallier’s classification^14^^,^^15^), (3) intact or reparable subscapularis tendon, and (4) those with a Hamada classification^17^ of grade 3 or lower. Forty patients were excluded as the following exclusion criteria: (1) those with severe cuff tear arthropathy (Hamada’s classification grade ≥4), (2) insufficient clinical records and preoperative and 1-year postoperative magnetic resonance imaging (MRI). Finally, a total of 28 patients were analyzed.

### Surgical technique

All operations were performed by an experienced shoulder surgeon (N.Y.) in the beach-chair position under general anesthesia. First, any lesions of the subscapularis were identified and repaired using suture anchors, if present. Long-head biceps tenodesis was performed if the tendon was subluxated or partially torn. Following acromioplasty, the posterosuperior cuff torn tendons were identified, and SCR was performed. At the same time, contralateral fascia lata was harvested. The graft was fixed to the superior glenoid using two 4.75-mm coiled-type anchors (Healicoil PK; Smith & Nephew, Andover, MA, USA) placed at the 1 o’clock and 11 o’clock positions. The 2 x 2 double-row transosseous-equivalent technique was used for lateral fixation at the greater tuberosity with suture anchors (SwiveLock; Arthrex, Naples, FL, USA), with the arm in 30° of abduction. The teres minor was repaired side-to-side with the graft posteriorly. The postoperative rehabilitation is descripted in supplementary I.

### Outcomes measurement

The muscle volume of the deltoid and teres minor was measured before and after surgery by an experience shoulder-specialized orthopedic surgeon with 5 years of experience (N.M.). Manual segmentation and muscle volume calculation were performed using the open-source medical image processing software 3D Slicer (3D Slicer, Earth, TX, USA).^12^ This software allows for visualization and analysis of medical imaging data, including segmentation and quantification of surface areas and volumes. Previous studies have reported that this software provides intraobserver reliability ranging from 0.78 to 0.95, interobserver reliability ranging from 0.85 to 0.98, and an accuracy of 99.8% for MRI volume measurements.^51^^,^^65^

**Figure 1:**
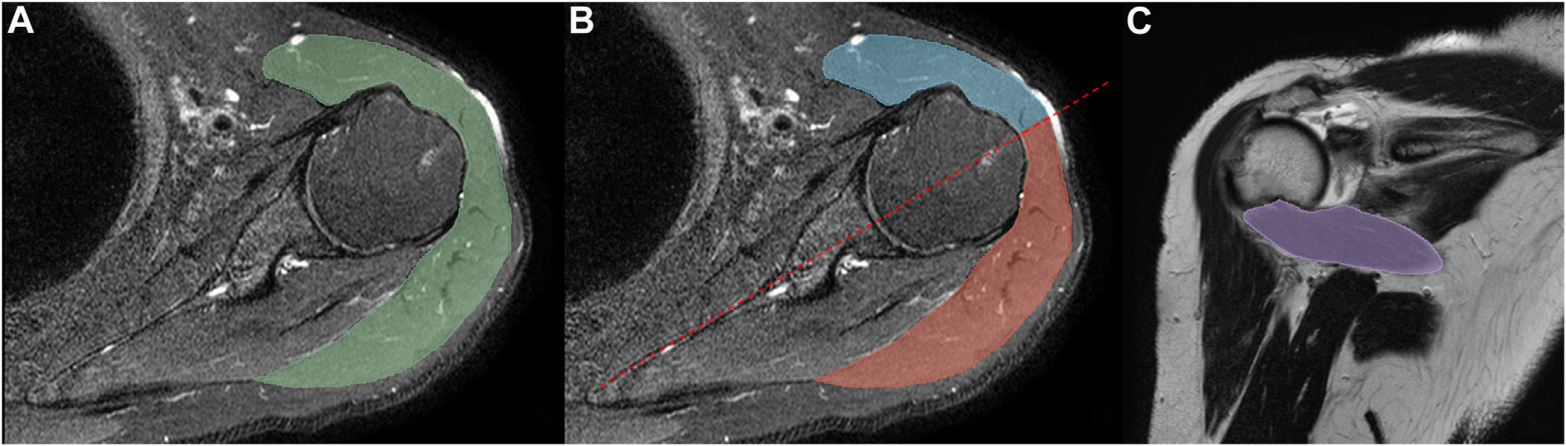
Illustrations demonstrating the segmentation in one shoulder. (**A**) Deltoid border (*green area*). (**B**) Anterior (*blue area*) and posterior deltoid (*red area*) were separated in the scapular plane (*red line*). (**C**) Teres minor border (*purple area*).

**Fig. 2:**
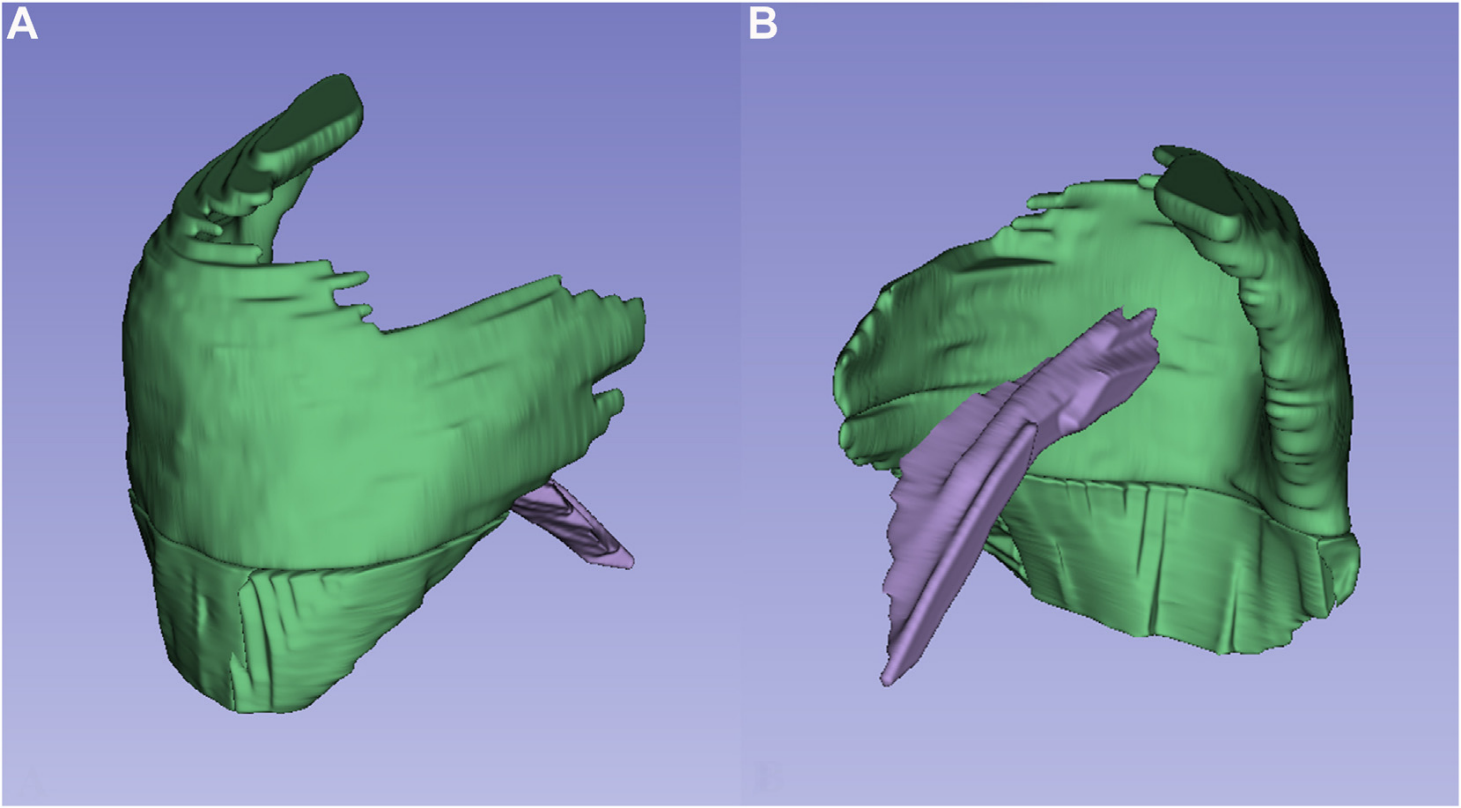
(**A, B**) The 3D-images of deltoid and teres minor muscles. *3D*, three dimensional.

MRI images were transferred to the software as DICOM files (Digital Imaging and Communications in Medicine). Based on T1-weighted, T2-weighted, and spectral attenuated inversion recovery images, the deltoid and teres minor muscles were segmented manually (Fig. 1). For deltoid muscle measurement, combined segmentation in axial, coronal, and sagittal series was used to gain maximum voxels (Fig. 2). For standardization, the deltoid volume was assessed from the upper end to the proximal insertion. After the entire deltoid muscle segmentation, we divided the deltoid into two parts (anterior and posterior) in the plane parallel to the scapular body and passing through the middle of the glenoid at the maximum humeral head size.

In previous literature, some studies divided the deltoid into three parts to measure the volume or area of each portion of the deltoid,^36^^,^^38^^,^^66^ while others divided it into two parts.^13^^,^^22^^,^^23^^,^^47^ Anatomically, there are reports describing that the deltoid can be divided into three parts—anterior, middle, and posterior—according to the location of their origin on the clavicle, acromion, and scapular spine, respectively.^55^ In MRI, these parts can be clearly identified only at the mid-glenoid level.^40^ However, it is not possible to accurately identify the fascia for splitting the deltoid along its entire length.^22^^,^^40^ This is why we chose the method of dividing it into two parts in this study.

The voxels anterior to this plane were defined as the anterior deltoid volume, and the voxels posterior to it were defined as the posterior deltoid volume. The entire teres minor, from its origin at the scapula to its insertion at the humeral head, was segmented. After segmentation, the volumes were calculated by converting the number of voxels to cubic centimeters. To assess muscle hypertrophy, the preoperative and 1-year postoperative muscle volumes of the deltoid and teres minor were compared.

Preoperative and 1-year postoperative shoulder angles and the strength of shoulder elevation and ER were reviewed. ER angle and muscle strength were assessed with the arm at the side position. The manual muscle testing (MMT) scale was used to represent muscle strength, which was determined on a scale from 0 to 5.^37^^,^^39^^,^^53^ For more accurate assessment, grades 1 to 5 were subdivided into 1-, 1, 1+, 2-, 2, 2+, …, 4+, 5-, and 5.^48^ Patients who had a postoperative MMT grade of 5 were defined as group A, while those with an MMT grade of 5- or below were defined as group B.

The muscle volume was compared between group A and group B. To reduce the effect of patients’ body size, we used the percentage of muscle volume change according to the following equation.

$$Percentage\;of\;volume\;change\;\left(\%\right)=\frac{postoperative\;volume-preoperative\;volume}{preoperative\;volume}\;x\;100$$

For fatty change evaluation, we used Goutallier’s classification.^14^ The relationship between preoperative and postoperative severity of teres minor fatty change, percentage of teres minor volume change, and ER angle and strength were evaluated.

### Statistical analysis

The data analysis was performed using SPSS 26 (IBM Corp., Armonk, NY, USA). To compare preoperative and postoperative variables, a paired t-test was used for the continuous variables analysis and Wilcoxon matched pairs test was used for the categorical variables analysis. Between group A and group B, independent t-test and Mann-Whitney U test were used for the continuous and categorical variables comparisons, respectively. Pearson, Spearman, or Kendall correlation analysis was used to evaluate the correlation between the variables: Pearson correlation is for the correlation between continuous and continuous variables, Spearman correlation is for the correlation between category and continuous variables, and Kendall correlation is for the correlation between category and category variables. The *P*-value of < .05 was defined as significant difference. Ten series of MRI were randomly reviewed by 2 raters and then inter and intra-observer reliability were analyzed using intraclass correlation coefficient (ICC). The observer agreement is classified as perfect agreement (ICC >0.80), substantial agreement (ICC = 0.61-0.80), moderate agreement (ICC = 0.41-0.60), fair agreement (ICC = 0.21-0.40), and slight agreement (ICC = 0-0.20).^34^

## Results

The mean age of the subjects at the time of surgery was 68 years (range, 51-83). There was a significant increase in the mean active shoulder elevation angle (*P* = .000), but no significant difference in the ER angle (*P* = .430) before and after surgery. Regarding the preoperative and postoperative MMT, the average postoperative elevation and ER MMT were significantly higher than the preoperative values (average elevation MMT: 4+ vs. 4-, W = −3.849, *P* = .000 and ER MMT: 5- vs. 4+, W = −3.332, *P* = .001) (Table I).

**Table I:** Differences between before and after surgery.

	Preoperative		Postoperative		*P*-value	95% CI		Reliability	
	Mean	SD	Mean	SD		Lower	Upper	Inter-observer	Intra-observer
Elevation angle (°)	110	45	144	22	.000	−51.2	−18.0		
Elevation MMT	4-		4+		.000∗				
External rotation (°)	32	18	34	17	.430	−8.9	3.9		
External rotation MMT	4+		5-		.001∗				
Deltoid volume (cm^3^)									
Total	267.3	74.1	267.6	73.0	.942	−9.6	8.9	0.97	0.98
Anterior	88.5	30.4	112.7	33.6	.000	−30.6	−17.7	0.97	0.99
Posterior	178.8	47.0	178.1	48.3	.840	−5.9	7.2	0.90	0.97
Teres minor volume (cm^3^)	20.9	8.3	24.6	10.3	.000	−5.4	−1.8	0.86	0.98

*MMT*, manual muscle testing (0-5); *CI*, confidence interval; *SD*, standard deviation.

∗ Wilcoxon matched pairs test.

According to the muscle segmentation, the intraclass correlation of inter and intraobserver reliability demonstrated perfect agreement (Table I). The muscle volume of the teres minor significantly increased postoperatively compared to preoperatively (24.6 cm³ vs. 20.9 cm³, respectively, *P* = .000). However, the posterior deltoid showed no significant differences (178.8 cm³ vs. 178.1 cm³, *P* = .84). The muscle volume of the teres minor increased more in patients with MMT grade 5 compared to those with MMT less than grade 5 (mean percentage of teres minor volume change: 22.3% vs. 9.4%, respectively, *P* = .074) (Table II).

**Table II:** Comparison between patients with ER MMT grade 15 and ER MMT less than 15.

	ER MMT grade 5 (N = 15)		ER MMT grade <5 (N = 13)		*P*-value	95% CI	
	Mean	SD	Mean	SD		Lower	Upper
Preoperative							
Elevation angle (°)	116	42	102	49	.409	−20.8	49.7
Elevation MMT	4		4-		.185∗		
ER (°)	37	17	25	17	.067	−0.9	25.6
ER MMT	5-		4+		.000∗		
Deltoid volume (cm^3^)							
Total	275.8	62.9	257.4	86.9	.523	−39.9	76.7
Anterior	94.2	25.9	81.9	34.8	.293	−11.2	35.9
Posterior	181.6	38.9	175.5	56.5	.741	−31.1	43.3
Teres minor volume (cm^3^)	21.7	7.5	19.9	9.4	.576	−4.7	8.3
Postoperative							
Elevation (°)	147	20	141	26	.500	−11.7	23.4
Elevation MMT	5-		4+		.046∗		
ERr (°)	41	18	27	13	.026	1.8	26.5
ER MMT	5		4+		.000∗		
Deltoid volume (cm^3^)							
Total	270.6	64.0	264.2	84.8	.821	−51.4	64.3
Anterior	115.4	29.8	109.6	38.4	.656	−20.7	32.3
Posterior	179.3	42.9	176.8	55.6	.896	−35.8	40.7
Teres minor volume (cm^3^)	26.6	10.4	22.2	10.1	.277	−3.6	12.3
Percentage of deltoid volume change (%)							
Total	−1.6	8.6	3.2	8.8	.154	−11.6	1.9
Anterior	18.1	12.1	26.1	14.2	.124	−18.1	2.3
Posterior	−1.2	9.8	1.1	8.8	.516	−9.6	4.9
Percentage of teres minor volume change (%)	22.30	17.74	9.42	18.78	.074	−1.3	27.0

*ER*, External rotation; *MMT*, Manual muscle testing (0-5); *CI*, confidence interval; *SD*, standard deviation.

∗ Mann-Whitney U test.

There was a weak positive correlation between the percentage change in teres minor volume and ER muscle strength (r = 0.308, *P* = .055). In contrast, the percentage change in posterior deltoid volume showed no correlation with ER MMT (r = −0.169, *P* = .195). The preoperative grading of fatty change in the teres minor, according to Goutallier’s classification, showed a weak negative correlation with 1-year postoperative ER MMT (r = −0.258, *P* = .065). However, there was a significant correlation between postoperative fatty change and ER MMT (r = −0.323, *P* = .028) (Table III). Additionally, the severity of teres minor fatty change before and after SCR had a negative correlation with the percentage change in teres minor volume (r = −0.298, *P* = .062, and r = −0.413, *P* = .015, respectively).

**Table III:** Correlation between the muscle volume, fatty change, and the 1-year postoperative ER angle and muscle strength.

	ER angle	ER MMT
Percentage of deltoid volume change (%)		
Total		
r	−0.80	−0.255
*P*-value	.342∗	.095†
95% CI	−1.0 to 0.245	−1.0 to 0.077
Anterior		
r	−0.056	−0.213
*P*-value	.389∗	.138†
95% CI	−1.0 to 0.267	−1.0 to 0.121
Posterior		
r	−0.027	−0.169
*P*-value	.445∗	.195†
95% CI	−1.0 to −0.293	−1.0 to 0.166
Percentage of teres minor volume change (%)		
r	0.170	0.308
*P*-value	.193∗	.055†
95% CI	−0.159 to 1.0	−0.02 to 1.0
Preoperative teres minor fatty change		
r	−0.234	−0.258
*P*-value	.116†	.065‡
95% CI	−1.0 to 0.1	−1.0 to −0.042
Postoperative teres minor fatty change		
r	−0.271	−0.323
*P*-value	.081†	.028‡
95% CI	−1.0 to 0.06	−1.0 to −0.113
	Teres minor fatty change	
	Preoperative	Postoperative
Percentage of teres minor volume change (%)		
r	−0.298	−0.413
*P*-value	.062†	.015†
95% CI	−1.0 to 0.031	−1.0 to −0.1

*ER*, external rotation; *MMT*, manual muscle testing (0-5); *CI*, confidence interval.

∗ Pearson correlation.

† Spearman correlation.

‡ Kendall correlation.

## Discussion

This study aims to identify the ER compensator in patients with massive irreparable rotator cuff tears who underwent SCR and recovered ER muscle strength by assessing muscle volume. Our data demonstrated a significant increase in teres minor muscle volume 1-year after surgery (mean 1-year postoperative volume: 24.6 cm³ vs. preoperative volume: 20.9 cm³, *P* < .001). The percentage change in teres minor volume showed a potential positive correlation with ER muscle strength and tended to be greater in patients with a postoperative ER MMT grade of 5 compared to those with a grade of less than 5. In contrast, the posterior deltoid did not show a significant increase in the volume compared to its preoperative state, and there was no correlation with ER muscle strength. According to our results, the teres minor is more likely to serve as the external compensator compared to the posterior deltoid.

Several biomechanical studies have reported that the infraspinatus, teres minor, and posterior deltoid contribute to ER moment arms.^16^^,^^21^^,^^31^ The infraspinatus has the longest ER moment arm when the arm is at the side position, while the teres minor has a longer moment arm at 90° abduction in the coronal plane, 90° abduction in the scapular plane, and 90° flexion position. On the other hand, the posterior deltoid is reported to have smaller ER moment arms in all of the mentioned positions.^31^ Previous electromyographic studies have shown that the infraspinatus, teres minor, and posterior deltoid exhibit increased electromyographic activity during shoulder ER with the arm at the side and the shoulder abduction position.^16^^,^^24^^,^^25^^,^^52^^,^^58^ The infraspinatus is most active during ER at lower levels of shoulder abduction position,^33^^,^^54^ while the teres minor^16^^,^^33^ and posterior deltoid^24^ demonstrate more activity at higher levels of shoulder abduction position, as indicated by electromyography^16^^,^^24^^,^^54^ and positron emission tomography studies.^33^ To our knowledge, there has been no direct comparison of electromyographic activity during ER between the teres minor and posterior deltoid.

Many previous studies have demonstrated that hypertrophy of the teres minor positively influences ER after surgery.^28^^,^^29^^,^^50^^,^^61^ Kikukawa et al^28^^,^^29^ reported that patients with posterosuperior rotator cuff tears who had a hypertrophic teres minor exhibited higher ER muscle strength after surgery compared to those with a normal or atrophic teres minor. Additionally, Paclot et al^50^ demonstrated that patients with a teres minor index of trophicity greater than 0.75 had a significantly higher ER angle with the arm at the side and at 90° of shoulder abduction position compared to those with an index less than 0.75. Thus, the teres minor is likely a key external rotator in their studies. Conversely, the role of the posterior deltoid remains controversial. Evidence both supports^69^ and refutes^38^^,^^66^ the contribution of the posterior deltoid to ER, with studies examining volume^38^^,^^69^ and cross-sectional area yielding mixed results.^66^

In the literature, some reports have investigated the relationship between ER angle^29^^,^^50^^,^^66^ or muscle strength^29^^,^^66^ and the cross-sectional area of the deltoid^20^^,^^66^ or teres minor.^20^^,^^28^^,^^29^^,^^50^^,^^61^ Other studies have examined the influence of deltoid muscle volume on ER after reverse total shoulder arthroplastyor arthroscopic rotator cuff repair.^38^^,^^59^^,^^69^ However, there are no reports that have quantitatively studied the relationship between muscle volume change and ER muscle strength after SCR. To our knowledge, this study is the first to address this issue.

Our results showed that the teres minor muscle experienced hypertrophy after surgery and had a potential correlation with ER muscle strength. The severity of teres minor fatty change negatively affected the percentage change in muscle volume after SCR. Additionally, our data indicated a negative correlation between both preoperative and postoperative severity of teres minor fatty change and ER muscle strength. These findings support the notion that a teres minor with less fatty change may increase muscle volume and better compensate for ER muscle strength after SCR.

There were several limitations to our study. First, accurately separating the deltoid muscle was challenging. As previously reported, the deltoid muscle comprises three parts.^55^ In single 2-dimensional MRI, it is relatively straightforward to identify the fascia of the deltoid to separate each part clearly at the mid-glenoid level.^40^ However, reliably identifying the planes distal to the mid-belly proved difficult.^22^ Consequently, we used a plane parallel to the scapular body passing through the center of the glenoid as an alternative based on the assumption that the muscle fibers posterior to the shoulder center of rotation are crucial for ER function.^6^^,^^18^^,^^26^

Second, our study only assessed ER in the arm at the side position. As a retrospective study, we had access only to data on ER with the arm at the side. We aimed to clarify which muscles compensate for ER in shoulders with a torn infraspinatus tendon. Theoretically, the infraspinatus is the most effective external rotator,^31^ particularly at lower levels of shoulder abduction,^33^ while the teres minor and posterior deltoid contribute at higher levels of shoulder abduction.^19^^,^^24^^,^^31^, ^32^, ^33^ Therefore, we think that ER with the arm at the side is a key indicator of compensation for the infraspinatus, more so than other arm positions.

Third, during the procedure, the torn infraspinatus was not reattached to its original footprint. We are uncertain whether the side-to-side suture connecting the teres minor and the graft affects mechanical changes in the teres minor. The teres minor might shift superiorly and align more closely with the infraspinatus, potentially enhancing ER at 0 degrees. This limitation highlights the need for further investigation.

Finally, our statistical analysis faced limitations due to the retrospective design based on imaging study. For instance, ER strength ideally should be measured with a dynamometer for precise quantitative data, rather than relying on MMT, which is more qualitative. Additionally, the small sample size only revealed trends rather than significant differences.

## Conclusions

Our results indicate that the teres minor has significant potential as a compensator for ER in patients with large to massive rotator cuff tears, including those involving the supraspinatus and infraspinatus tendons, following SCR. Additionally, the degree of fatty change in the teres minor serves as a crucial predictor of postoperative improvements in ER muscle strength.

## Acknowledgments

The authors would like to acknowledge the help and support given by all the officers in the Department of Orthopaedic Surgery, Tohoku University School of Medicine.

## Disclaimers:

Funding: No funding was disclosed by the authors.

Conflicts of interest: All authors, their immediate family, and any research foundation with which they are affiliated have not received any financial payments or other benefits from any commercial entity related to the subject of this article.
