# Supplementary material for: Which muscle is the external rotation compensator after superior capsular reconstruction?
Source: JSES Int. 2024 Sep 27;9(1):123–9. doi: 10.1016/j.jseint.2024.09.010 (PMC11784471; doi:10.1016/j.jseint.2024.09.010)
Supplement: Supplementary material [file mmc1.docx]

**Postoperative rehabilitation**

All patients received supervised, individualized rehabilitation once or twice a week for at least 6 months. The arm was immobilized in abduction and internal rotation using an abduction brace for 8 weeks. Isometric deltoid muscle exercises were started at 1 week. At 4 weeks, passive shoulder range-of-motion exercises including external rotation exercises were started. Active-assisted shoulder range-of-motion exercises were initiated in the supine position at 6 weeks. The arm was free to move for activities of daily living, and active-assisted exercises were initiated with patients in the sitting position at 8 weeks. Muscle strengthening exercises such as cuff exercise using a rubber band were started after 12 weeks.
